# Protection from killed whole-cell cholera vaccines: a systematic review and meta-analysis

**DOI:** 10.1016/S2214-109X(25)00107-X

**Published:** 2025-06-25

**Authors:** Hanmeng Xu, Amanda Tiffany, Francisco J Luquero, Suman Kanungo, Godfrey Bwire, Firdausi Qadri, Daniela Garone, Louise C Ivers, Elizabeth C Lee, Espoir Bwenge Malembaka, Vincent Mendiboure, Malika Bouhenia, Lucy Breakwell, Andrew S Azman

**Affiliations:** a Department of Epidemiology, Johns Hopkins Bloomberg School of Public Health, Baltimore, MD, USA; b Department of Epidemiology of Microbial Diseases, Yale School of Public Health, New Haven, CT, USA; c Global Immunization Division, Centers for Disease Control and Prevention, Atlanta, GA, USA; d Gavi, the Vaccine Alliance, Geneva, Switzerland; e National Institute for Research in Bacterial Infections, Indian Council on Medical Research, Kolkata, India; f Uganda Ministry of Health, Kampala, Uganda; g icddrb, Dhaka, Bangladesh; h Médecins Sans Frontières International, Geneva, Switzerland; i Massachusetts General Hospital Center for Global Health, Boston, MA, USA; j Harvard University Global Health Institute, Boston, MA, USA; k Center for Tropical Diseases and Global Health, Université Catholique de Bukavu, Bukavu, Democratic Republic of the Congo; l WHO, Geneva, Switzerland; m Geneva Centre for Emerging Viral Diseases and Division of Tropical and Humanitarian Medicine, Geneva University Hospitals, Geneva, Switzerland

## Abstract

**Background:**

Killed whole-cell oral cholera vaccines (kOCVs) are a standard prevention and control measure in cholera-endemic areas and during outbreaks and humanitarian emergencies. New evidence has emerged and the ways in which the vaccines are used have changed. We aimed to provide an updated synthesis of evidence on protection conferred by kOCV.

**Methods:**

In this systematic review and meta-analysis, we used the same search procedure as a previous systematic review to identify randomised clinical trials (RCTs) and observational studies that reported estimates of protection conferred by kOCVs against medically attended, confirmed cholera. Eligible studies in English, French, Spanish, or Chinese published up until March 8, 2024, including those identified in the previous review, were included. Data on efficacy and effectiveness were extracted, as were the number of doses, duration of follow-up, and age group. Efficacy and effectiveness estimates were summarised separately using random-effect models to estimate protection by time since vaccination; meta-regression models were used to estimate protection, by dose, as a function of time since vaccination. This updated study is registered along with the original review with PROSPERO (CRD42016048232).

**Findings:**

We identified 8205 records published online up until March 8, 2024, including 6224 articles from the previous review and 1981 articles from our new search (after Jan 1, 2016). Of these, 53 were eligible for full-text review. Five RCTs and ten observational studies from 23 publications were included. Average two-dose efficacy 12 months after vaccination was 55% (95% CI 46–62), declining to 44% (25–59) 48 months after vaccination. Average two-dose effectiveness was 69% (58–78) 12 months after vaccination, declining to 47% (9–70) 48 months after vaccination. Only one RCT assessed one-dose efficacy and found sustained protection for 24 months (57% [42–69]) among those 5 years and older with no significant protection in younger children. Average one-dose effectiveness 12 months after vaccination was 60% (51–68) and after 24 months was 47% (34–58). Using age group-specific meta-analysis, we found that average two-dose efficacy in children younger than 5 years was half that of older individuals.

**Interpretation:**

Two doses of kOCV provide protection against medically attended cholera for at least 4 years after vaccination. One dose of kOCV provides protection for at least 2 years after vaccination, but wanes faster than that of two doses. Children younger than 5 years are less protected by kOCVs than those aged 5 years and older, regardless of the number of doses received.

**Funding:**

Bill & Melinda Gates Foundation.

## Introduction

Killed whole-cell oral cholera vaccines (kOCV) are part of the standard cholera control and prevention package often used in combination with water, sanitation, and hygiene improvements. kOCVs have been used reactively to control outbreaks, where short-term vaccine protection is most crucial, and pre-emptively in areas with endemic cholera, where longer-term vaccine protection is key.

Two versions of modern kOCVs first underwent clinical trials in the 1980s: a simple kOCV and another with an added cholera toxin recombinant B-subunit.^1^ Both kOCVs were further developed, but today, vaccines with the B-subunit are used only for travellers. All vaccines used in public health programmes in cholera-affected countries, including those used preventively and for outbreak response through the global stockpile, are WHO-approved (prequalified) kOCVs, including both primary serotypes of the seventh pandemic *Vibrio cholerae* O1. As of 2024, four biologically similar kOCVs have been approved by WHO: Shanchol in 2011 (Sanofi Pasteur, Lyon, France); Euvichol in 2015 (EuBiologics, Seoul, South Korea); Euvichol-Plus in 2017 (EuBiologics); and Euvichol-S in 2024 (EuBiologics).

Clinical trials and observational studies in several cholera-endemic countries such as Bangladesh, Haiti, and India have shown that kOCVs are safe and immunogenic, although estimates of protection vary widely across studies.^2^, ^3^, ^4^, ^5^ Vaccine-derived protection wanes over time, although how quickly immunity wanes remain unclear. A 2017 systematic review and meta-analysis estimated two-dose efficacy against medically attended cholera of 58% (95% CI 42–69) and field effectiveness of 76% (62–85), with protection lasting at least 3 years.^6^ Few studies, however, tracked outcomes beyond 2 years. Protection was lower in children younger than 5 years, although few studies provided age-stratified estimates, and differences in epidemiological setting were not accounted for.

**Research in context**

**Evidence before this study**

Killed whole-cell oral cholera vaccines (kOCVs) are one of the standard cholera control and prevention tools. The Global Taskforce for Cholera Control's Oral Cholera Vaccine Working Group published a systematic review and meta-analysis of kOCV protection against medically attended cholera in 2017, estimating an average two-dose efficacy from randomised controlled trials of 58% (95% CI 42–69; *I*^2^ 58%) and two-dose effectiveness from observational studies of 76% (62–85; *I*^2^ 0%). One other review on clinical protection from kOCVs, published in 2018, specifically looked at the effectiveness of kOCV for outbreak response and estimated an effectiveness against medically attended cholera of 75% (61–84) after vaccination with at least one dose.

**Added value of this study**

In the 7 years since the previous review was published, kOCV availability has changed, new evidence on kOCV protection has accumulated, and since 2022, constrained kOCV supply has restricted outbreak response to single-dose campaigns. Compared with the previous review, this systematic review and meta-analysis includes estimates from three additional observational studies, initiated after 2017, which used Euvichol/Euvichol-Plus (EuBiologics)—the only available vaccines in the global stockpile during most of 2024. New estimates of one-dose protection from one clinical trial and four observational studies with follow-up of up to 4 years after vaccination and new estimates of two-dose protection from three observational studies with follow-up of at least 2 months are also included. This review also presents estimates of protection as a number that varies over time to reflect waning immunity using both stratified models and meta-regression.

**Implications of all the available evidence**

Two doses of kOCV provide substantial protection against medically attended cholera for at least 4 years. One dose of kOCV provides protection for at least 2 years, which lends support to the current one-dose outbreak response policy of WHO, for which short-term protection is key. As new simplified derivatives of Euvichol (eg, Euvichol-S; approved in April, 2024) are approved by WHO and become the predominant kOCVs available, documenting protection conferred by these vaccines over time by age group and epidemiological setting will be important. This review highlights important questions that remain to be addressed when designing future studies and kOCV vaccination programmes, including the magnitude and duration of different dosing regimens in settings with different historical incidence rates of cholera.

Countries can apply for kOCV for outbreak response and preventive campaigns, with Gavi, The Vaccine Alliance covering vaccine and operational costs for most cholera-affected regions. Since the inception of the global stockpile, demand has exceeded supply.^7^, ^8^ In response to numerous requests for vaccines, the International Coordinating Group (comprised UNICEF, Médecins sans Frontières, WHO, and the International Federation of Red Cross and Red Crescent Societies), which oversees allocation of the global stockpile for outbreak response, temporarily switched to a one-dose strategy for outbreak response in October, 2022.^9^, ^10^ Consequently, no two-dose preventive campaigns occurred in 2023, 2024, or the first quarter of 2025 (time of final writing). Furthermore, in 2023, Sanofi ceased production of Shanchol, the vaccine for which most evidence on protection has been generated. At the time of writing, two kOCVs are available through the stockpile: Euvichol-Plus (with the same bacterial strains as Shanchol) and Euvichol-S (a subset of strains).^11^, ^12^ These vaccines were licensed on the basis of immunological studies, not clinical outcomes (vaccine comparisons in appendix pp 2–3).

With new data on WHO-approved kOCVs, evolving usage of these vaccines, and a rising global kOCV demand, an up-to-date review of protection by dose, population, and setting is needed to guide outbreak response and revaccination timelines in endemic areas. This study updates a previous systematic review and meta-analysis, offering detailed insights on protection by doses, time since vaccination, and age.

## Methods

### Literature search and data abstraction

We searched PubMed, Embase, Scopus, ISI Web of Science, and the Cochrane Review Library for literature published from Jan 1, 2016, to March 8, 2024, using the search terms from a previous systematic review.^6^ Results were merged with the previous review covering studies up to Jan 1, 2016 (appendix p 3). Records were imported to a web-based screening tool (https://www.covidence.org/) for automatic deduplication. Experts from the Global Task Force on Cholera Control were consulted to identify missing publications. The original 2016 review was pre-registered in PROSPERO (CRD42016048232).

Titles and abstracts were independently screened by two of three reviewers (HX, ASA, and AT), following inclusion and exclusion criteria from the previous review.^6^ Conflicts were resolved by a third reviewer or through discussion (HX, ASA, and AT). We included clinical trials or observational studies published in English, Spanish, French, or Chinese that used medically attended, confirmed cholera cases (with at least one diagnostic test for *V cholerae* O1/O139) to estimate kOCV efficacy or effectiveness. Both new studies and follow-up of previous studies were included. Study location, timeframe, and dosing regimen were recorded to assess publication independence. Duplicates from the previous review were excluded from the new search.

During full-text review, we extracted data on the study setting, target population, study type, vaccine, dosing regimen, case-confirmation method, method or methods of vaccination status ascertainment, estimates of vaccine protection, and measures of uncertainty. Vaccine protection estimates include efficacy, determined in randomised (clinical) trials done in rigorously controlled conditions, and effectiveness, determined in observational studies done in more real-world settings. Efficacy estimates are less susceptible to confounding and selection bias than effectiveness estimates. When available, we extracted several estimates of vaccine protection, including those disaggregated by age, time since vaccination, and number of doses received. We combined the data extracted from the eligible studies identified in both the previous and the new search for all analyses. Data from studies on kOCVs with recombinant B-subunit were extracted but excluded from analyses because of their restricted use in travellers. We only extracted published estimates and did not calculate new protection estimates from manuscript data.

### Risk of bias assessment

Two reviewers (HX and AT) independently assessed the risk of bias for each study (both new and old) using the Newcastle-Ottawa Scale for observational studies and the Cochrane Collaboration's tool for randomised controlled trials (RCTs). Conflicts were resolved through discussion or a third reviewer (ASA). The assessment relied solely on methods described in the publications. If one study had more than one publication for different follow-up periods, all were considered in the bias assessment.

### Data analysis

Following Bi and colleagues,^6^ we used reported vaccine efficacy or effectiveness estimates and 95% CIs to calculate standard errors. For estimates with one-sided CIs, two-sided CIs were reconstructed.^6^, ^13^ Given the waning of kOCV protection, we analysed efficacy and effectiveness over time using time-bin stratified analyses and using meta-regression.

For stratified analyses, estimates were grouped by the midpoint of the follow-up period (eg, 0–12 months, 12–24 months, 24–36 months, 36–48 months, and 48–60 months after vaccination). For each time bin, we estimated a pooled mean vaccine efficacy or effectiveness using a random-effects model, with an empirical Bayes estimator for the between-study variance, and assessed heterogeneity using the *I*^2^ statistic.^14^, ^15^

Stratified protection estimates combine data from slightly different follow-up periods (ie, within the 0–12 month time period, some estimates cover only the first 3 months, whereas others span the entire period) and are not constrained to remain constant or decrease over time. To refine this, we used meta-regression to model protection continuously over time since vaccination, incorporating estimates on the basis of their specific time ranges. We fit mixed-effects meta-regression models using the natural logarithm of time since vaccination as the primary fixed effect and estimated between-study variance with a restricted maximum likelihood estimator via the R package *metafor*.^15^ We report 95% CIs for mean protection and 95% prediction intervals to show the expected variability in future studies across settings. We compared different meta-regression models using time transformations and random slopes, evaluated by Akaike information criterion. The midpoint of each estimate's period was used, with separate models by dose and study type (effectiveness and efficacy). We used leave-one-out analyses to assess the influence of individual datapoints on waning estimates.

To compare vaccine protection in children younger than 5 years to that in those aged 5 years and older, we extracted age-stratified efficacy and effectiveness estimates. These estimates were pooled by age group and doses to estimate mean vaccine efficacy or effectiveness.

### Role of the funding source

The funder of the study had no role in the study design, data collection, data analysis, data interpretation, or writing of the report.

## Results

**Figure 1:**
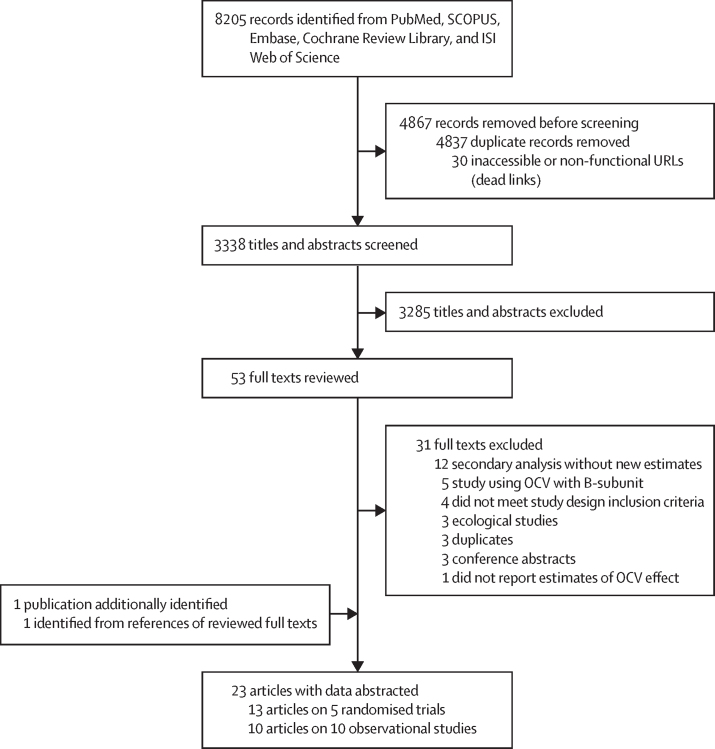
PRISMA flow chart of the record screening process The screening process is shown for the combined dataset of records identified from the 2016 and 2023–24 searches. OCV=oral cholera vaccine.

We identified 8205 records published online up until March 8, 2024, including 6224 articles from the previous review and 1981 articles from our new search (after Jan 1, 2016; figure 1). Of these, 53 were eligible for full-text review. One additional publication eligible for full-text review was identified from a reference review of identified publications.^16^ Five publications were excluded given that they used a kOCV with the cholera-toxin-B subunit.^17^, ^18^, ^19^, ^20^, ^21^ 23 publications met the data abstraction inclusion criteria, with estimates from five RCTs (13 publications)^1^, ^2^, ^3^, ^4^, ^22^, ^23^, ^24^, ^25^, ^26^, ^27^, ^28^, ^29^, ^30^ and ten observational studies (ten publications;^5^, ^16^, ^31^, ^32^, ^33^, ^34^, ^35^, ^36^, ^37^, ^38^ figure 1, table 1; appendix p 6).

**Table 1:** Overview of clinical trials and observational studies that met the inclusion criteria

	Study initiation year*	**Study location**	**Study design**	**Vaccine**	**Comparison treatment**	**Number of doses for primary outcome**	**Study population**	**Follow-up duration after vaccination**
**Efficacy studies (clinical trials)**								
Clemens et al,^1^ Clemens et al,^2^ van Loon et al,^24^ Clemens et al,^25^ and Clemens et al^26^	1985	Matlab, Bangladesh	Individually randomised controlled trial	WC†	Same vial with heated killed *Escherichia coli* K12 strain	Three	Children aged 2–15 years and all women aged >15 years, non-pregnant	48 months
Trach et al^28^	1992	Hue, Viet Nam	Household randomised trial without placebo	WC†	Without placebo	Two	Individuals aged ≥1 year	10 months
Sur et al,^4^ Bhattacharya et al,^22^ and Sur et al^23^	2006	Kolkata, India	Cluster-randomised controlled trial	Shanchol	Same vial with heated killed *E coli* K12 strain	Two	Non-pregnant individuals aged ≥1 year	60 months
Qadri et al^3^ and Ali et al^30^	2011	Dhaka, Bangladesh	Cluster-randomised trial without placebo	Shanchol	Without placebo	Two	Non-pregnant individuals aged ≥1 year	48 months*
Qadri et al^27^ and Qadri et al^29^	2014	Dhaka, Bangladesh	Cluster-randomised trial	Shanchol	Same vial with inert placebo agent	One	Non-pregnant individuals aged ≥1 year, without history of an oral cholera vaccine	24 months*
**Effectiveness studies (observational studies)**								
Wierzba et al^31^	2011	Puri District, India (preventive)	Case-control	Shanchol	Medically attended, test-negative controls	Two	Non-pregnant individuals aged ≥1 year	34 months
Ivers et al^5^	2012	Artibonite Department, Haiti (reactive)	Case-control	Shanchol	Age-matched and neighbourhood-matched community controls	Two	Individuals aged ≥1 year	22 months
Luquero et al^32^	2012	Boffa and Forecariah Districts, Guinea (reactive)	Case-control	Shanchol	Age-matched and neighbourhood-matched community controls	Two	Individuals aged ≥1 year	4 months
Franke et al^34^‡	2012 and 2014†	Artibonite Department and Central Department, Haiti (reactive)	Case-control	Shanchol	Age-matched and neighbourhood-matched community controls	One and two	Non-pregnant individuals aged ≥1 year	48 months
Azman et al^33^	2015	Juba, South Sudan (reactive)	Case-cohort	Shanchol	Age-matched and neighbourhood-matched community controls	One	Individuals aged ≥1 year	2 months
Ferreras et al^16^‡	2016	Lusaka, Zambia (reactive)	Case-control	Shanchol	Age-matched and neighbourhood-matched community controls	One	Individuals aged >1 year	2 months
Grandesso et al^35^‡	2016	Lake Chilwa, Malawi (reactive)	Case-control	Shanchol	Medically attended, test-negative controls	Two	Individuals aged >1 year (83% were adult fishermen)	3 months
Sialubanje et al^36^‡	2017	Lusaka, Zambia (reactive)	Case-control	Euvichol-Plus	Community-matched age, sex, and neighbourhood controls	Two	Individuals aged ≥1 year	6 months
Matias et al^38^‡	2018	Mirebalais, Haiti (preventive)	Case-control	Euvichol	Age-matched, gender-matched and neighbourhood-matched community controls	Two	Individuals aged ≥1 year	24 months
Malembaka et al^37^‡	2020	Uvira, Democratic Republic of the Congo (reactive)	Case-control	Euvichol-Plus	Age-matched, gender-matched and neighbourhood-matched community controls	One	Individuals aged ≥1 year	36 months

WC=whole cell.

* Year when the first vaccination occurred.

† Precursor to Dukoral (without B subunit) and Shanchol, with similar antigenic composition.

‡ New follow-up estimates not included in Bi and colleagues.^6^

The five RCTs estimating vaccine efficacy were done in Kolkata (India),^4^, ^22^, ^23^ Matlab (Bangladesh),^1^, ^2^, ^24^, ^25^, ^26^ Dhaka (Bangladesh),^3^, ^27^, ^29^, ^30^ and Hue (Viet Nam),^28^ with the earliest trial starting in 1985 in Matlab^1^, ^2^, ^24^, ^25^, ^26^ and the most recent trial starting in 2014 in Dhaka.^27^, ^29^ One trial reported efficacy for a three-dose regimen,^1^, ^2^, ^24^, ^25^, ^26^ three for a two-dose regimen,^3^, ^4^, ^22^, ^23^, ^28^, ^30^ and one for a one-dose regimen.^27^, ^29^ Follow-up time after vaccination ranged from 10 months to 5 years for two-dose or three-dose trials, and 2 years for the sole one-dose trial. The two earliest trials used vaccine formulations that were precursors to the current kOCVs.^1^, ^2^, ^24^, ^25^, ^26^, ^28^ The three trials in Kolkata^4^, ^22^, ^23^ and Dhaka^3^, ^27^, ^29^, ^30^ used Shanchol. None of these trials used Euvichol or Euvichol-Plus. Four trials reported that case finding was done only through passive surveillance in clinics where cholera cases were identified and treated, whereas the Matlab trial also did active community-based case finding.^1^ Outcomes in all trials were based on culture confirmation. Two studies had a low risk of bias across all study-quality domains;^23^, ^27^ one had low risk across all but two study domains, for which the risk was unclear.^1^ The remaining two studies had a high risk of bias in a few domains, including those related to masking of participants, outcomes, and allocation concealment (appendix p 7).^3^, ^28^

The ten observational studies included nine case-control studies and one case-cohort study. They had a wider geographical range than the trials, with six from sub-Saharan Africa, one from Asia, and three from the Caribbean. Seven were initiated after vaccination campaigns done in response to an outbreak and three were done pre-emptively in endemic areas, including Puri (India),^31^ Artibonite and Central Department (Haiti),^5^, ^34^ and Uvira (the Democratic Republic of the Congo).^37^ Two studies were done after one-dose emergency vaccination campaigns.^16^, ^33^ The follow-up period after vaccination ranged from 3 months to 4 years for two-dose estimates, and 2 months to 4 years for one-dose estimates. Seven studies used Shanchol, one study used Euvichol,^38^ and two studies used Euvichol-Plus.^36^, ^37^ All observational studies identified cases through passive clinical surveillance. Four studies used culture alone to classify cholera cases in their main analyses,^5^, ^31^, ^34^, ^36^ two used PCR alone,^35^, ^38^ one used culture and PCR,^16^ and three used a combination of PCR, culture, and rapid diagnostic tests.^32^, ^33^, ^37^ Nine studies ascertained vaccination status on the basis of self-report and reference to a vaccination card if available, and one study relied solely on an electronic vaccination registry.^31^ Availability of vaccination cards varied across studies, ranging from 0% to 82%, with a median of 50% (IQR 19–60). All ten observational studies had a low risk of selection bias; one had a low risk of bias related to comparability and most had moderate-to-high risk of bias related to ascertainment of exposure due to self-reporting of vaccination status. All nine case-control studies did not report adequate data on non-response rates (appendix p 3).

**Figure 2:**
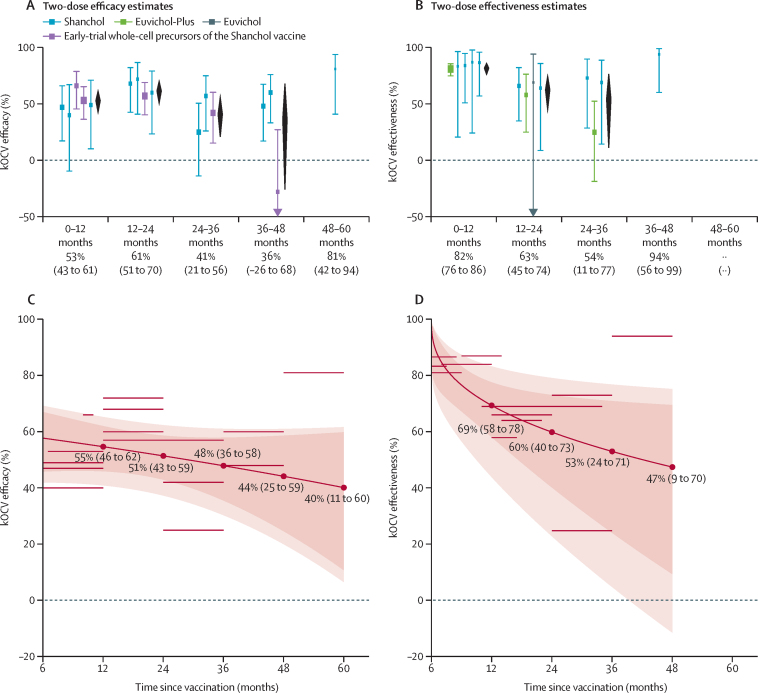
Stratified and meta-regression estimates of the efficacy and effectiveness of two doses of kOCV as a function of time since vaccination The upper panels show stratified estimates of efficacy (A) and effectiveness (B) by time since vaccination. The trial done in Matlab, Bangladesh used three doses of kOCV but was also included.^24^ Estimates are grouped into the five follow-up duration categories by the midpoint of the time window during which the estimate was measured. Bars and squares show 95% CIs and point estimates, respectively, of efficacy or effectiveness for each study, coloured by vaccine type. Black diamonds are the estimated pooled efficacy or effectiveness and 95% CI by follow-up period, with numerical values shown beneath the x-axis. If there is only one estimate in the follow-up period, the estimate from the study is presented on the x-axis. Estimates of *I*^2^ for the pooled estimates in panel A are 0% (0–12 months), 0% (12–24 months), 25% (24–36 months), and 81% (36–48 months). Estimates of *I*^2^ for the pooled estimates in panel B are 36% (0–12 months), 0% (12–24 months), and 52% (24–36 months). The bottom panels illustrate meta-regression results for mean two-dose efficacy (C) and effectiveness (D) as a function of time since vaccination, with the shaded envelope representing the 95% CIs (darker region) and 95% prediction intervals (lighter region). The horizontal grey lines represent the data from the literature that were used to fit the meta-regression models; the length of the line indicates the duration of follow-up (months since vaccination) and the line's position on the y-axis marks the magnitude of the point estimate (%). The dashed horizontal line at y=0 denotes no protective effect (0%) of kOCV. kOCV=killed whole-cell oral cholera vaccine.

We identified 16 efficacy estimates (five publications reporting on four trials)^3^, ^22^, ^24^, ^28^, ^30^ and 13 effectiveness estimates (eight publications reporting on eight observational studies)^5^, ^31^, ^32^, ^34^, ^35^, ^36^, ^37^, ^38^ for two kOCV doses (figure 2A, B; appendix pp 4–5). Efficacy estimates covered 0–12 months (five estimates), 12–24 months (four estimates), 24–36 months (three estimates), 36–48 months (three estimates), and 48–60 months (one estimate) after vaccination (appendix pp 4–5). Effectiveness estimates covered 0–12 months (five estimates), 12–24 months (four estimates), 24–36 months (three estimates), and 36–48 months (one estimate) after vaccination (appendix pp 4–5). Efficacy estimates ranged from 40% (95% CI –10 to 67) to 66% (46 to 79) in the first year (0–12 months), 57% (42 to 70) to 72% (42 to 87) in the second year (12–24 months), 25% (–13 to 51) to 57% (26 to 75) in the third year (24–36 months), and from less than zero to 60% (33 to 76) in the fourth year (36–48 months; figure 2A; appendix pp 4–5). Effectiveness estimates ranged from 81% (72 to 84) to 87% (32 to 98) in the first year, 58% (27 to 76) to 69% (–71 to 94) in the second year, and 25% (–19 to 52) to 73% (30 to 90) in the third year; in the one study estimating in the fourth year, effectiveness was 94% (56 to 99; figure 2B; appendix pp 4–5).

Given waning protection, we fit meta-regression models to estimate two-dose protection over time (figure 2C, D). Two-dose efficacy was 55% (95% CI 46 to 62) at 12 months, 51% (43 to 59) at 24 months, 48% (36 to 58) at 36 months, 44% (25 to 59) at 48 months, and 40% (11 to 60) at 60 months (figure 2C). Effectiveness estimates were numerically higher than efficacy estimates in the first 2 years (figures 2C, D). Two-dose effectiveness was 69% (58 to 78) at 12 months, 60% (40 to 73) at 24 months, 53% (24 to 71) at 36 months, and 47% (9 to 70) at 48 months. Leave-one-study-out meta-regression analyses illustrated that the two longest efficacy^22^, ^24^ and effectiveness studies^34^, ^37^ influenced protection estimates, especially in the fourth and fifth years (appendix p 8). Sensitivity analyses excluding estimates on pre-Shanchol vaccines^24^, ^28^ showed similar results (appendix p 12).

**Figure 3:**
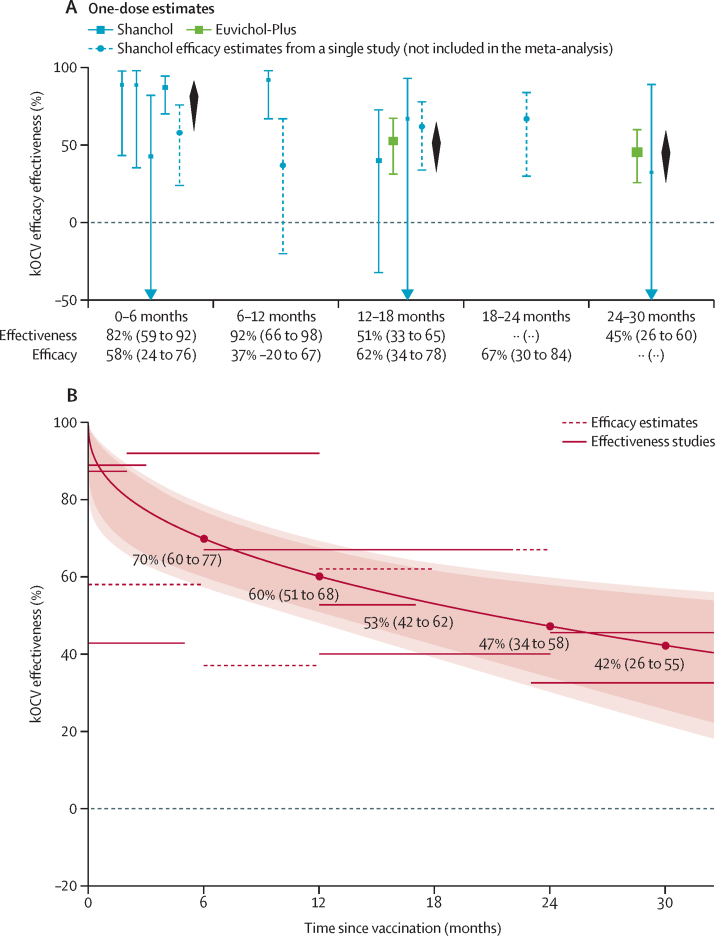
Stratified and meta-regression estimates of the efficacy and effectiveness of one dose of kOCV as a function of time since vaccination (A) Stratified estimates of efficacy or effectiveness by 6-month time periods after vaccination. Bars and squares show 95% CIs and point estimates, respectively, of efficacy (dashed lines) or effectiveness (solid lines), coloured by vaccine type. Black diamonds are the estimated pooled effectiveness and 95% CI by follow-up period, with numerical values beneath the x-axis in black (first row). If there is only one effectiveness estimate in the follow-up period, the estimate from that study is presented. Efficacy estimates for those 5 years and older.^29^ Estimates of *I*^2^ for the pooled estimates in panel A are 36% (0–6 months), 0% (12–18 months), and 0% (24–30 months). (B) Meta-regression results for mean one-dose effectiveness as a function of time since vaccination, with the shaded envelope representing the 95% CIs (darker region) and 95% prediction intervals (lighter region). The horizontal grey lines represent the data from the literature that were used to fit the meta-regression models; the length of the line indicates the duration of follow-up (months since vaccination), and the position of the line on the y-axis marks the magnitude of the point estimate (%). The dashed horizontal line at y=0 denotes no protective effect (0%) of kOCV. kOCV=killed whole-cell oral cholera vaccine.

We identified four efficacy estimates (one publication reporting on one trial)^29^ and ten effectiveness estimates (eight publications reporting on eight observational studies)^5^, ^16^, ^31^, ^32^, ^33^, ^34^, ^35^, ^37^ reported for one kOCV dose (figure 3A; appendix p 5). Efficacy estimates covered 0–6 months, 6–12 months, 12–18 months, and 18–24 months after vaccination (appendix p 5). Effectiveness estimates included 0–6 months (four studies), 6–12 months (one study), 12–18 months (three studies), and 24–30 months (two studies). The RCT measuring one-dose efficacy found sustained protection among those 5 years and older for 2 years. In that trial, one-dose efficacy among those 5 years and older was 58% (95% CI 24 to 76) at 0–6 months, 37% (–20 to 67) at 6–12 months, 62% (34 to 78) at 12–18 months, and 67% (30 to 84) at 18–24 months. One-dose effectiveness estimates for 0–6 months and 6–12 months ranged from 43% (–84 to 82) to 92% (66 to 98). For 12–18 months after vaccination, the three effectiveness estimates ranged from 40% (–31 to 73) to 67% (–62 to 93). The two one-dose effectiveness estimates covering 24–30 months were 46% (26 to 60) and 32% (–318 to 89).

Meta-regression analyses showed similar effectiveness between one-dose and two-dose regimens within the first year, with faster decay for one-dose protection (appendix p 9). One-dose effectiveness was 70% (60 to 77) at 6 months, decreasing to 60% (51 to 68) at 12 months, 53% (42 to 62) at 18 months, 47% (34 to 58) at 24 months, and 42% (26 to 55) at 30 months (figure 3B). Leave-one-study-out analyses yielded similar results (appendix p 10).

Five trials and two observational studies that used two or three doses of kOCV reported age-stratified estimates (table 2). Across all studies reporting age-stratified two-dose estimates, protection was consistently lower in children younger than 5 years, except in a study in Viet Nam. Across studies with age-stratified estimates, the pooled two-dose efficacy for children younger than 5 years was 31% (95% CI 14 to 45; *I*^2^=0%), with a 36-month weighted mean follow-up period, compared with 62% (49 to 71; *I*^2^=60%) for participants aged 5 years and older, across a 37-month weighted mean follow-up period (appendix p 11).

**Table 2:** kOCV effectiveness and efficacy by age group and dose

	**Doses**	**Study design**	**Location**	**Follow-up time (months)**	**VE (95% CI)**			
					<5 years	≥5 years	5–15 years	≥15 years
Clemens et al (1990)^2^	Three	Randomised	Bangladesh	36	23 (1 to 43)	68 (59 to 76)*	NA	NA
Ivers et al (2015)^5^	Two	Observational	Haiti	22	50 (−850 to 97)	72 (36 to 88)	NA	NA
Franke et al (2018)^35^	Two	Observational	Haiti	48	28 (−109 to 75)	77 (58–88)	NA	NA
Trach et al (1997)^29^	Two	Randomised	Viet Nam	10	68 (14 to 88)	66 (42 to 80)	NA	NA
Sur et al (2011)^4^	Two	Randomised	India	36	43 (7 to 68)	NA	88 (71 to 96)	61 (37 to 78)
Qadri et al (2015)^3^	Two	Randomised	Bangladesh	24	44 (−35 to 77)	NA	33 (−94 to 77)	56 (31 to 72)
Ali et al (2021)^31^	Two	Randomised	Bangladesh	48	24 (−30 to 56)	49 (35 to 60)	NA	NA
Malembaka et al (2024)^38^	One	Observational	Democratic Republic of the Congo	36	50 (16 to 70)	48 (33 to 60)	NA	NA
Franke et al (2018)^35^	One	Observational	Haiti	48	−69 (−786 to 68)	97 (70 to 100)	NA	NA
Qadri et al (2016)^28^	One	Randomised	Bangladesh	6	16 (−49 to 53)	NA	63 (−39 to 90)	56 (16 to 77)
Qadri et al (2018)^30^	One	Randomised	Bangladesh	24	−13 (−68 to 25)	NA	52 (8 to 75)	59 (42 to 71)

kOCV=killed whole-cell oral cholera vaccine. VE=vaccine efficacy (for randomised studies) and vaccine effectiveness (for observational studies).

* VE for both sexes aged 5–15 years and for only women older than 15 years.

Evidence for one-dose protection among young children remains scarce. One randomised trial in Bangladesh and two case-control studies, one each in the Democratic Republic of the Congo and Haiti, reported estimates for children younger than 5 years (table 2; appendix p 11). The trial in Bangladesh found that protection among children younger than 5 years at 6 months after vaccination was 16% (–49 to 53) and at 24 months after vaccination was –13% (–68 to 25).^27^, ^29^ By comparison, protection among those aged 5 years and older was 57% (24 to 76) at 6 months after vaccination and 58% (42 to 69) at 24 months after vaccination.^27^, ^29^ By contrast, the case-control study in the Democratic Republic of the Congo found nearly identical estimates of protection across the two age groups, reporting 50% (16 to 70) protection in children younger than 5 years and 48% (33 to 60) in those aged 5 years and older 12–36 months after vaccination.^37^

## Discussion

This review summarises data from five RCTs and ten observational studies across ten countries, showing that the two-dose kOCV regimen provides substantial protection for at least 4 years. A single dose offers protection for at least the first 2 years after vaccination, but protection probably wanes faster than it does after two doses (appendix p 9). We found that evidence on protection in populations with little to no historical exposure to *V cholerae* O1 remains scarce, and vaccine protection is notably lower in children aged 1–4 years.

Since the last review in 2017, cholera vaccine use has grown, both in terms of the number of doses and the number of countries conducting campaigns. Although 2022 and 2023 saw a shift to nearly exclusive use of the vaccine for outbreak response, cholera-endemic countries are also planning for more sustained use of kOCV.^39^ Several important questions on how to design vaccination programmes remain: where to vaccinate; when to revaccinate; the number of doses to use (one *vs* two, especially if revaccinating); the optimal timing between doses; and whether recommendations should be age-group specific. The 2017 WHO position paper on cholera vaccines states that populations should not be revaccinated within a 3-year period.^40^ Our results suggest that, at least in populations frequently exposed to *V cholerae* O1, protection from two doses is sustained over this period, with evidence from both randomised and observational studies demonstrating protection well beyond 3 years, in the fourth and fifth years after vaccination. Only short-term estimates of protection, within the first year,^16^, ^33^ are available from settings that had not reported cholera for several years, so the duration of vaccine protection in immunologically naive populations remains unclear.

In 2022, because of a global kOCV shortage, the International Coordinating Group, the body that makes allocation decisions for the emergency stockpile of kOCVs, temporarily suspended use of two-dose regimens.^10^ Our analysis suggests that a one-dose strategy is effective for short-term outbreak control,^41^ providing protection for at least 2 years in the general population. However, data are scarce for populations with little previous exposure to pandemic *V cholerae*. Results from a trial in Bangladesh showed minimal protection for children younger than 5 years,^27^ in contrast to a case-control study from the Democratic Republic of the Congo,^37^ which found similar levels of protection in the first 2 years after vaccination regardless of age group. The reasons for this discrepancy are unclear; however, one potential explanation is that indirect effects from vaccinated household members in each study differed, with the randomised trial less subject to the influence of indirect effects from household members.^42^, ^43^

Our review has several limitations, including reliance on non-standardised time intervals and differences between vaccine compositions. Most evidence comes from Shanchol, which is no longer produced. Among currently available kOCVs, three estimates from observational studies of Euvichol and Euvichol-Plus have been published, with no estimates for Euvichol-S. We did not have enough data to reliably detect differences in protection between different vaccines, although the point estimates from Euvichol and Euvichol-Plus are generally consistent with previous evidence from other vaccines (figure 2, 3). Sensitivity analyses excluding pre-Shanchol vaccines showed largely unchanged effectiveness estimates, but efficacy estimates with modern kOCVs indicated no waning over the first 5 years (appendix p 12).

With the exception of one study,^33^ all effectiveness studies used a case-control design, most with notable risk of bias in terms of the ascertainment of exposure (usually through self-report) and in the comparability between cases and controls. Future evidence using more rigorous prospective designs, including rigorous vaccination ascertainment, longer duration of follow-up after vaccination, and causal inference methods, could help improve confidence in estimates and potentially allow for regulatory approvals without the need for randomised trials.^44^ Evaluating protection in special populations, such as people who are immunocompromised and those who are malnourished, is also crucial.

Our review was not able to address several pertinent policy-relevant questions related to vaccine use, including the optimal timing between doses, and when, and if, vaccination with one dose should be followed up with the full two-dose schedule. These decisions should consider not only the direct protection from the vaccine, but other epidemiological and operational considerations to ensure doses will have an effect.

Our pooled effectiveness estimates were higher than pooled efficacy estimates over the first 2 years. Although the reasons for this finding are probably multifactorial, we hypothesise that it could in part be explained by the fact that the RCTs were largely done in highly endemic communities, where the average age of infection is lower than in other areas. By contrast, much of the observational data comes from less endemic communities. The median percentage of cases younger than 5 years in randomised trials was 27·8%, compared to 16·7% in observational studies (appendix p 13). Given that kOCV provides less protection for young children, we might expect to have lower estimates of protection in areas with a higher proportion of young children as cases. Another potential reason for this discrepancy is the potential effect of indirect effects on the estimates of effectiveness.^45^

More than 31 countries have used kOCVs to control cholera in the past decade.^39^ The absence of clear evidence on the duration of protection from one dose and two doses, combined with the global kOCV shortage, have complicated efforts to sustain vaccine-derived protection. Our synthesis reconfirms that two doses offer substantial protection, at least into the fifth year after vaccination, whereas one dose can provide protection over at least the first 2 years. This evidence supports the current policy of using one dose in outbreak response, in which short-term protection is most crucial. It is unclear whether this level of protection would be sustained in populations that are largely immunologically naive and whether a single-dose strategy is efficient, particularly in endemic areas with high ongoing risk for cholera.^10^ Our review illustrates the temporal scale of waning direct protection, but indirect protection might wane even faster in some settings because of human mobility patterns.^3^, ^46^

While the world waits for universal access to water and sanitation in addition to better and more cholera vaccines, improving vaccination-campaign quality and enhancing cholera surveillance are essential to effectively manage and reduce cholera risk.

### Contributors

### Data sharing

All data extracted in the review process and code needed to reproduce analyses are available at https://github.com/HopkinsIDD/kOCV-review.

## Declaration of interests
