# Supplementary material for: Protection from killed whole-cell cholera vaccines: a systematic review and meta-analysis
Source: Lancet Glob Health. 2025 Jun 25;13(7):e1203–12. doi: 10.1016/S2214-109X(25)00107-X (PMC12208782; doi:10.1016/S2214-109X(25)00107-X)
Supplement: Supplementary appendix [file mmc1.pdf]

THE LANCET
Global Health

Supplementary appendix

This appendix formed part of the original submission and has been peer reviewed. We post it as supplied by the authors.

Supplement to: Xu H, Tiffany A, Luquero FJ, et al. Protection from killed whole-cell cholera vaccines: a systematic review and meta-analysis. *Lancet Glob Health* 2025; **13:** e1203–12.

Protection from killed whole-cell cholera vaccines: a systematic review and meta-analysis

Hanmeng Xu, Amanda Tiffany*, Francisco J Luquero, Suman Kanungo, Godfrey Bwire, Firdausi Qadri, Daniela Garone, Louise C Ivers, Elizabeth C Lee, Espoir Bwenge Malembaka, Vincent Mendiboure, Malika Bouhenia, Lucy Breakwell, Andrew S Azman*

Supplementary Appendix

Table of Contents

Figure S1. **PRISMA flow chart of screening process of newly identified records in 2024.** 6
Figure S2. **Risk of bias summary for clinical trials following the Cochrane Collaboration Tool.** 7
Figure S3. **Efficacy (A) and effectiveness (B) of two-dose kOCVs from leave-one-study-out analysis.** 8
Figure S4. **Comparison of estimated effectiveness of one-dose and two-dose kOCV over time since vaccination predicted by meta-regression models.**. 9
Figure S5. **Effectiveness of one-dose kOCVs from leave-one-study-out analysis** 10
Figure S6. **Pooled efficacy of two-dose whole-cell kOCV by age group.**. 11
Figure S7. **Stratified and meta-regression estimates of the efficacy and effectiveness of two doses of killed whole-cell OCV (kOCV) as a function of time since vaccination, after removing vaccines that came before Shanchol vaccines.** 12
Figure S8. **Distribution of percentage of cholera cases under 5 years old in efficacy studies and effectiveness studies.** 13

Table S1. **Comparison of the killed whole-cell oral cholera vaccines (kOCV).** 2
Table S2. **Search terms and search results.** 3
Table S3. **Risk of bias summary for included observational studies.**. 3
Table S4. **Point estimates of two-dose efficacy or effectiveness of whole-cell OCV included in the meta-analysis.** 4
Table S5. **Point estimates of one-dose efficacy or effectiveness of whole-cell OCV included in the meta-analysis.**. 5

Table S1. ***Comparison of the killed whole-cell oral cholera vaccines (kOCV).***

Product name	Shanchol	Euvichol	Euvichol-Plus	Euvichol-S
Manufacturer	Sanofi Pasteur	EuBiologics	EuBiologics	EuBiologics
Composition	*V cholerae* O1 Inaba classical strain Cairo 48 [300 lipopolysaccharide ELISA unit (LEU)] - heat inactivated<br><br>*V cholerae* O1 Ogawa classical strain Cairo 50 [300 LEU] - heat inactivated<br><br>*V cholerae* O1 Inaba El Tor strain Phil 6973 [600 LEU] - formalin inactivated<br><br>*V cholerae* O1 Ogawa Classical strain Cairo 50 [300 LEU] - formalin inactivated<br><br>*V cholerae* O139 4260B [600 LEU] - formalin inactivated	*V cholerae* O1 Inaba classical strain Cairo 48 [300 lipopolysaccharide ELISA unit (LEU)] - heat inactivated<br><br>*V cholerae* O1 Ogawa classical strain Cairo 50 [300 LEU] - heat inactivated<br><br>*V cholerae* O1 Inaba El Tor strain Phil 6973 [600 LEU] - formalin inactivated<br><br>*V cholerae* O1 Ogawa Classical strain Cairo 50 [300 LEU] - formalin inactivated<br><br>*V cholerae* O139 4260B [600 LEU] - formalin inactivated	*V cholerae* O1 Inaba classical strain Cairo 48 [300 lipopolysaccharide ELISA unit (LEU)] - heat inactivated<br><br>*V cholerae* O1 Ogawa classical strain Cairo 50 [300 LEU] - heat inactivated<br><br>*V cholerae* O1 Inaba El Tor strain Phil 6973 [600 LEU] - formalin inactivated<br><br>*V cholerae* O1 Ogawa Classical strain Cairo 50 [300 LEU] - formalin inactivated<br><br>*V cholerae* O139 4260B [600 LEU] - formalin inactivated	*V cholerae* O1 Inaba El Tor strain Phil 6973 [900 LEU] - formalin inactivated<br><br>*V cholerae* O1 Ogawa Classical strain Cairo 50 [600 LEU] - formalin inactivated
Time of WHO prequalification	2011	2015	2017	2024
Packaging	Glass vials	Glass vials	Plastic tubes	Plastic tubes

Production	Discontinued in 2023	Currently in production

Table S2. ***Search terms and search results.*** *We did two rounds of search in five databases. The first round of search was on January 22, 2023. We did a second round of search on March 8, 2024 to include studies published between the two searches.*

Engine	Language restriction	Exact Search Query	Number of records on Jan 22, 2023	Number of records on Mar 8, 2024
Pubmed	None	cholera*[Title/Abstract] AND (vaccin*[Title/Abstract]) AND (effect*[Title/Abstract] OR efficacy[Title/Abstract] OR protect*[Title/Abstract])	577	98
Embase	None	cholera*:ab,ti AND vaccin*:ab,ti AND (efficacy:ab,ti OR effect*:ab,ti OR protect*:ab,ti)	637	144
Scopus	None	TITLE-ABS(cholera*) AND TITLE-ABS(vaccin*) AND TITLE-ABS(efficacy OR effect* OR protect*) AND NOT INDEX (embase)	146	119
ISI Web of Science	None	TI=(cholera* AND vaccin*) AND TS=(efficacy OR effect* OR protect*)	227	24
Cochrane Review Library	None	cholera* AND vaccin* AND (efficacy OR effect* OR protect*)	0	9

Table S3. ***Risk of bias summary for included observational studies.*** *The maximum number of stars (indicating lowest risk of bias) is indicated next to MAX for each criterion. Assessment is based on the Newcastle-Ottawa Scale for observational studies [1].*

CASE-CONTROL STUDIES	SELECTION MAX ****	COMPARABILITY MAX **	EXPOSURE MAX ***
Wierzba et al. 2015	***	*	**
Ivers et al. 2015	****	*	**
Luquero et al. 2014	****	**	*
Franke et al. 2018	****	*	*
Ferreras et al. 2018	****	*	*
Grandesso et al. 2019	***		*
Sialubanje et al. 2022	****	*	
Malembaka et al. 2023	****		*
Matias et al. 2023	****	*	*
**CASE-COHORT STUDIES**	**SELECTION MAX ******	**COMPARABILITY MAX ****	**OUTCOME MAX *****
Azman et al. 2016	****	*	**

Table S4. ***Point estimates of two-dose efficacy or effectiveness of whole-cell OCV included in the meta-analysis.***

Estimate type	Follow-up duration group	Location	Actual follow-up time (months)	VE (95% CI)	Study
Efficacy	0-12 months	Bangladesh	[0, 12]	47 (17-66)	**Ali et al, 2021** [2]
		India	[0, 12]	40 (-10-67)	Bhattacharya et al, 2013 [3]
		Vietnam	[8, 10]	66 (46-79)	Trach et al, 1997 [4]
		Bangladesh	[1, 12]	53 (38-66)	van Loon et al, 1996 [5]
		Bangladesh	[0, 12]	49 (10-71)	Qadri et al, 2015 [6]
	12-24 months	Bangladesh	[12, 24]	68 (42-82)	**Ali et al, 2021** [2]
		India	[12, 24]	72 (42-87)	Bhattacharya et al, 2013 [3]
		Bangladesh	[12, 24]	57 (42-70)	van Loon et al, 1996 [5]
		Bangladesh	[12, 24]	60 (23-79)	Qadri et al, 2015 [6]
	24-36 months	Bangladesh	[24, 36]	25 (-13-51)	**Ali et al, 2021** [2]
		India	[24, 36]	57 (26-75)	Bhattacharya et al, 2013 [3]
		Bangladesh	[24, 36]	42 (18-62)	van Loon et al, 1996 [5]
	36-48 months	Bangladesh	[36, 48]	48 (16-67)	**Ali et al, 2021** [2]
		India	[36, 48]	60 (33-76)	Bhattacharya et al, 2013 [3]
		Bangladesh	[36, 48]	-28 (-114-31)	van Loon et al, 1996 [5]
	48-60 months	India	[48, 60]	81 (42-94)	Bhattacharya et al, 2013 [3]
Effectiveness	0-12 months	Zambia	[0, 6]	81 (72-84)	**Sialubanje et al, 2022** [7]
		Malawi	[0, 3]	83 (21-96)	**Grandesso et al, 2019** [8]
		Haiti	[2, 12]	84 (53-95)	**Franke et al, 2018** [9]
		Haiti	[6, 14]	87 (32-98)	Ivers et al, 2015 [10]
		Guinea	[0, 5]	87 (57-96)	Luquero et al, 2014 [11]
	12-24 months	Haiti	[12, 24]	66 (34-82)	**Franke et al, 2018** [9]
		Haiti	[14, 22]	64 (10-86)	Ivers et al, 2015 [10]
		Haiti	[10, 27]	69 (-71-94)	Matias et al, 2023 [12]

		DRC	[12, 17]	58 (27-76)	**Malembaka et al. 2024** [13]
	24-36 months	Haiti	[24, 36]	73 (30-90)	**Franke et al, 2018** [9]
		India	[23, 34]	69 (14-89)	Wierzba et al, 2015 [14]
		DRC	[24, 36]	25 (-19, 52)	**Malembaka et al. 2024** [13]
	36-48 months	Haiti	[36, 48]	94 (56-99)	**Franke et al, 2018** [9]

Table S5. ***Point estimates of one-dose efficacy or effectiveness of whole-cell OCV included in the meta-analysis.*** *The four estimates of one-dose OCV efficacy (Qadri et al. 2018 [16]) were not included in the meta-analysis as they are the only efficacy estimates available.*

Estimate Type	Follow-up duration group	Location	Actual follow-up duration (months)	VE (95%CI)	Study
Efficacy	0-6 months	Bangladesh	[0, 6]	58 (24-76)	Qadri et al, 2018[16]
	6-12 months	Bangladesh	[6, 12]	37 (-20-67)	Qadri et al, 2018[16]
	12-18 months	Bangladesh	[12, 18]	62 (34-78)	**Qadri et al, 2018** [16]
	18-24 months	Bangladesh	[18, 24]	67 (30-84)	Qadri et al, 2018[16]
Effectiveness	0-6 months	Zambia	[0, 2]	89 (43-98)	**Ferreras et al, 2018** [17]
		Malawi	[0, 3]	89 (36-98)	**Grandesso et al, 2019** [8]
		Guinea	[0, 5]	43 (-84-82)	Luquero et al, 2014 [11]
		South Sudan	[0, 2]	87 (70-100)	Azman et al, 2016 [18]
	6-12 months	Haiti	[2, 12]	92 (66-98)	**Franke et al, 2018** [9]
	12-18 months	Haiti	[12, 24]	40 (-31-73)	**Franke et al, 2018** [9]
		DRC	[12, 17]	53 (31-67)	**Malembaka et al. 2024** [13]
		Haiti	[6, 22]	67 (-62-93)	Ivers et al, 2015 [10]
	24-30 months	DRC	[24, 36]	46 (26-60)	**Malembaka et al. 2024** [13]
		India	[23, 34]	32 (-318-89)	Wierzba et al, 2015 [14]

SECTION 2. SUPPLEMENTARY FIGURES.

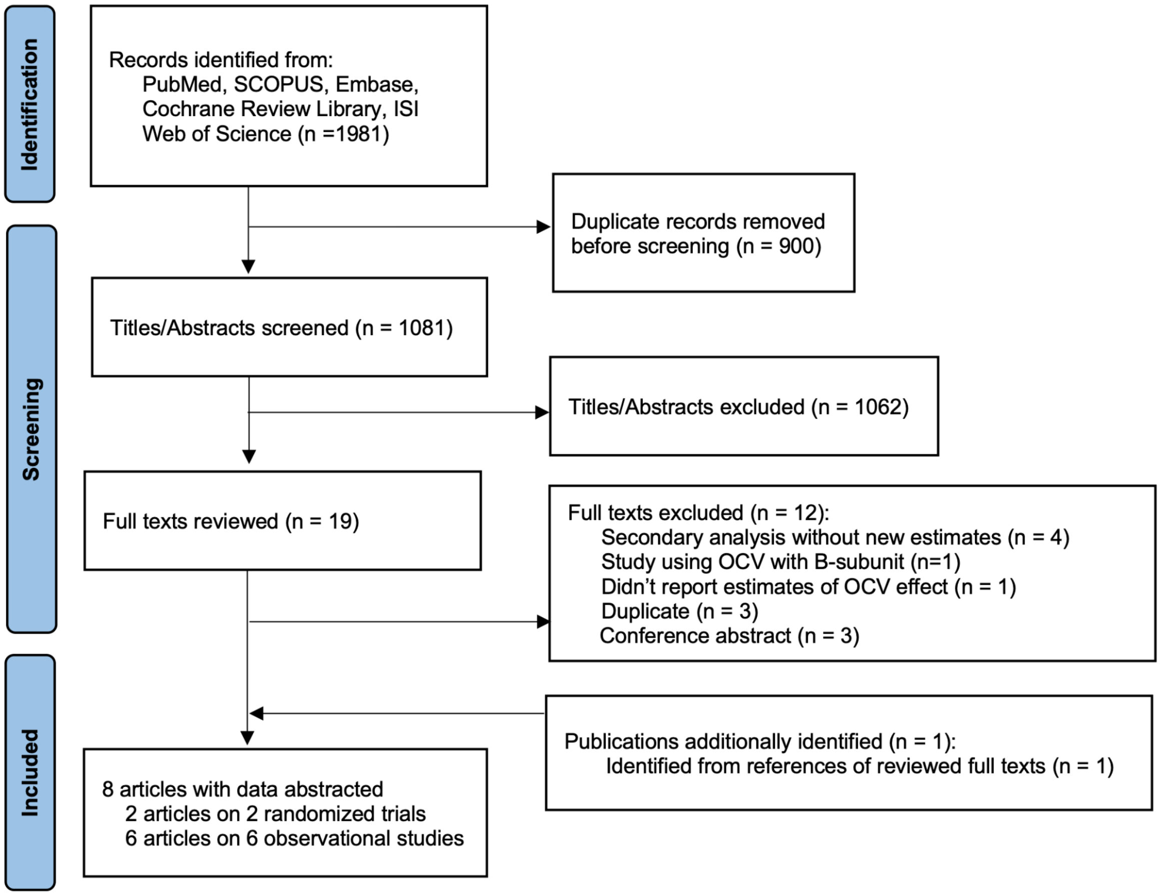

Figure S1. ***PRISMA flow chart of screening process of newly identified records in 2024.*** *This flow chart illustrates the screening process for records identified in the new search, with date of publication restricted to January 1st, 2016 to March 8th, 2024.*

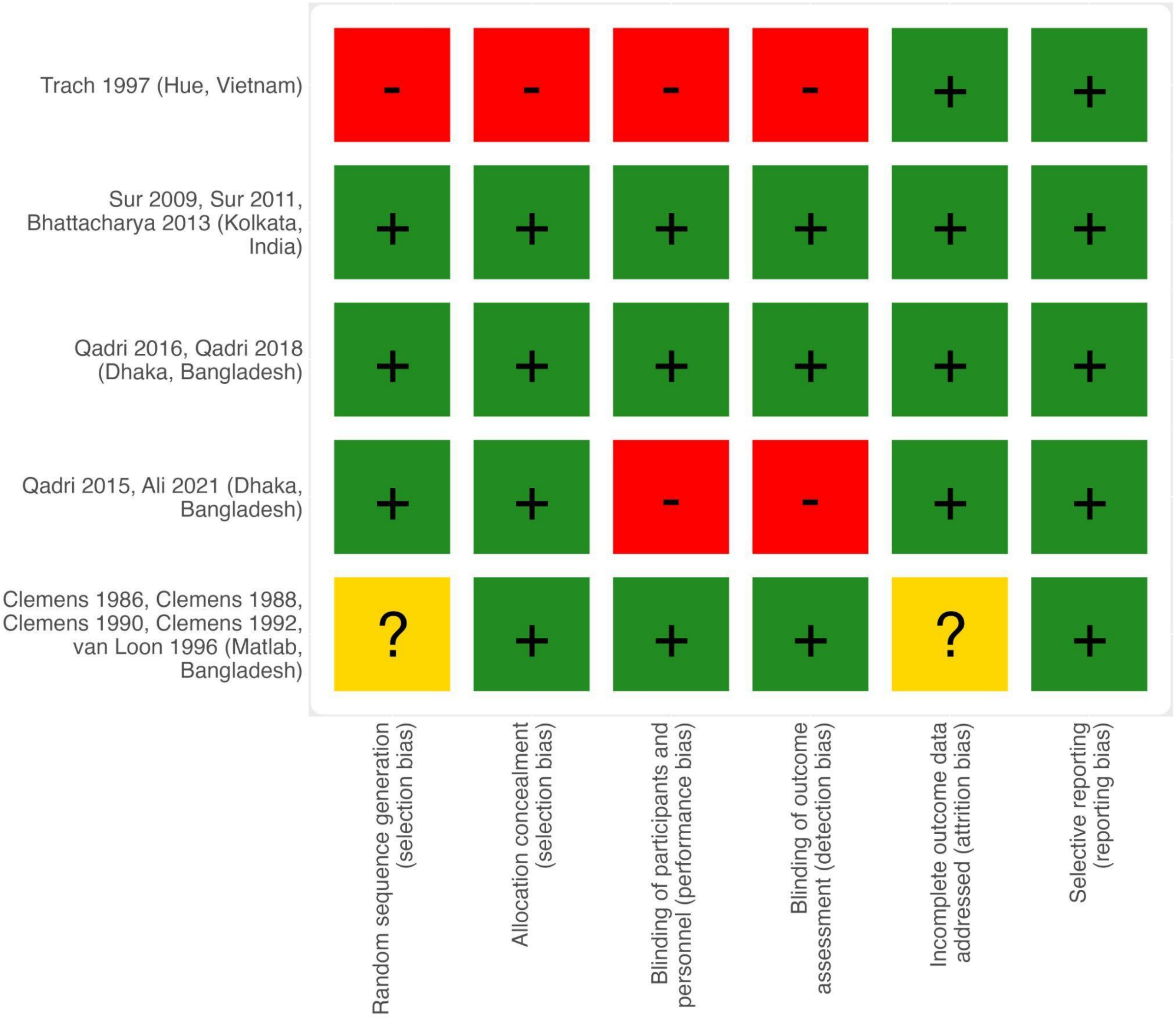

Figure S2. ***Risk of bias summary for clinical trials following the Cochrane Collaboration Tool.*** *Green cells represent low risk of bias, yellow cells indicate unclear risk of bias and red cells indicate high risk of bias.*

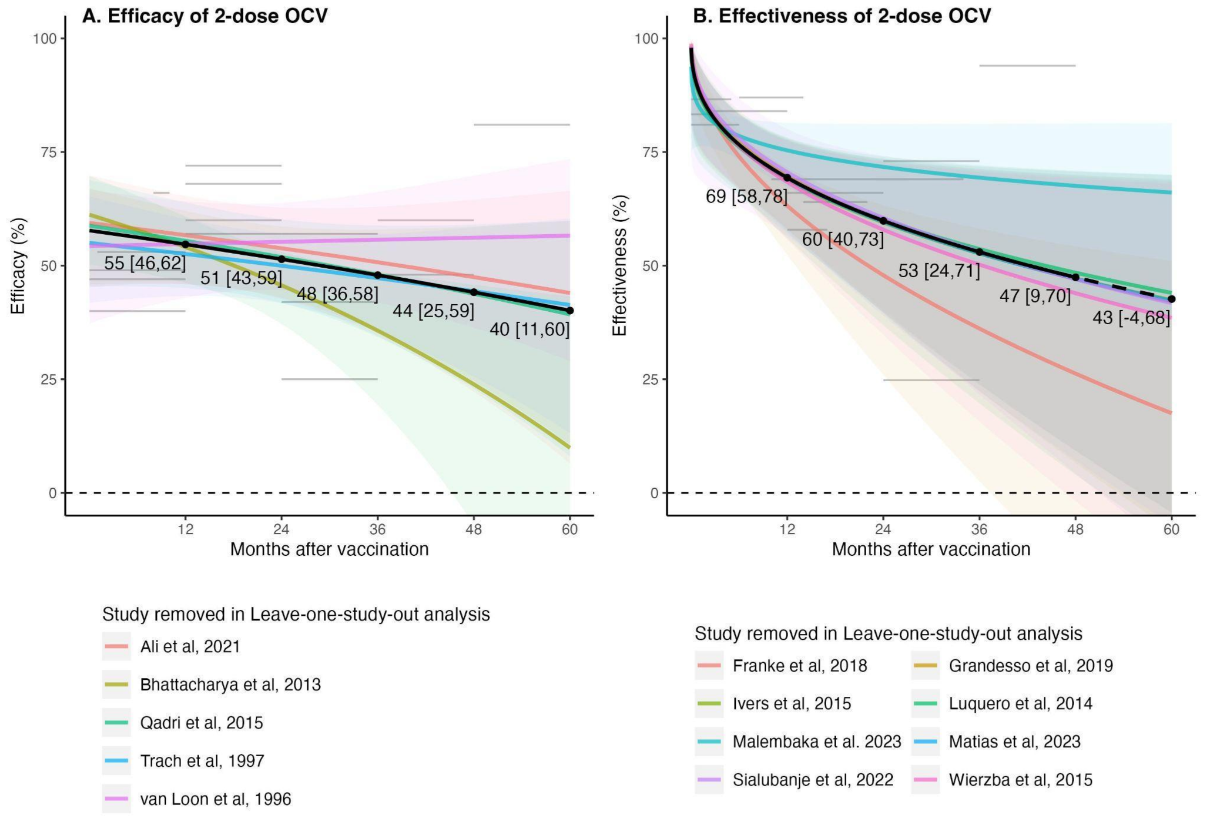

Figure S3. ***Efficacy (A) and effectiveness (B) of two-dose kOCVs from leave-one-study-out analysis.*** *The black solid lines and labels are estimated efficacy or effectiveness using the full dataset of all two-dose estimates. The colored lines represent estimated efficacy and effectiveness after leaving out estimates from one study. The horizontal gray lines represent the full dataset that was used to fit the meta-regression models, the length of the line indicates the duration of follow-up (months since vaccination). The line's position on the y-axis marks the magnitude of the point estimate (%). The dashed horizontal line at y=0 denotes no protective effect (0%) of kOCV.*

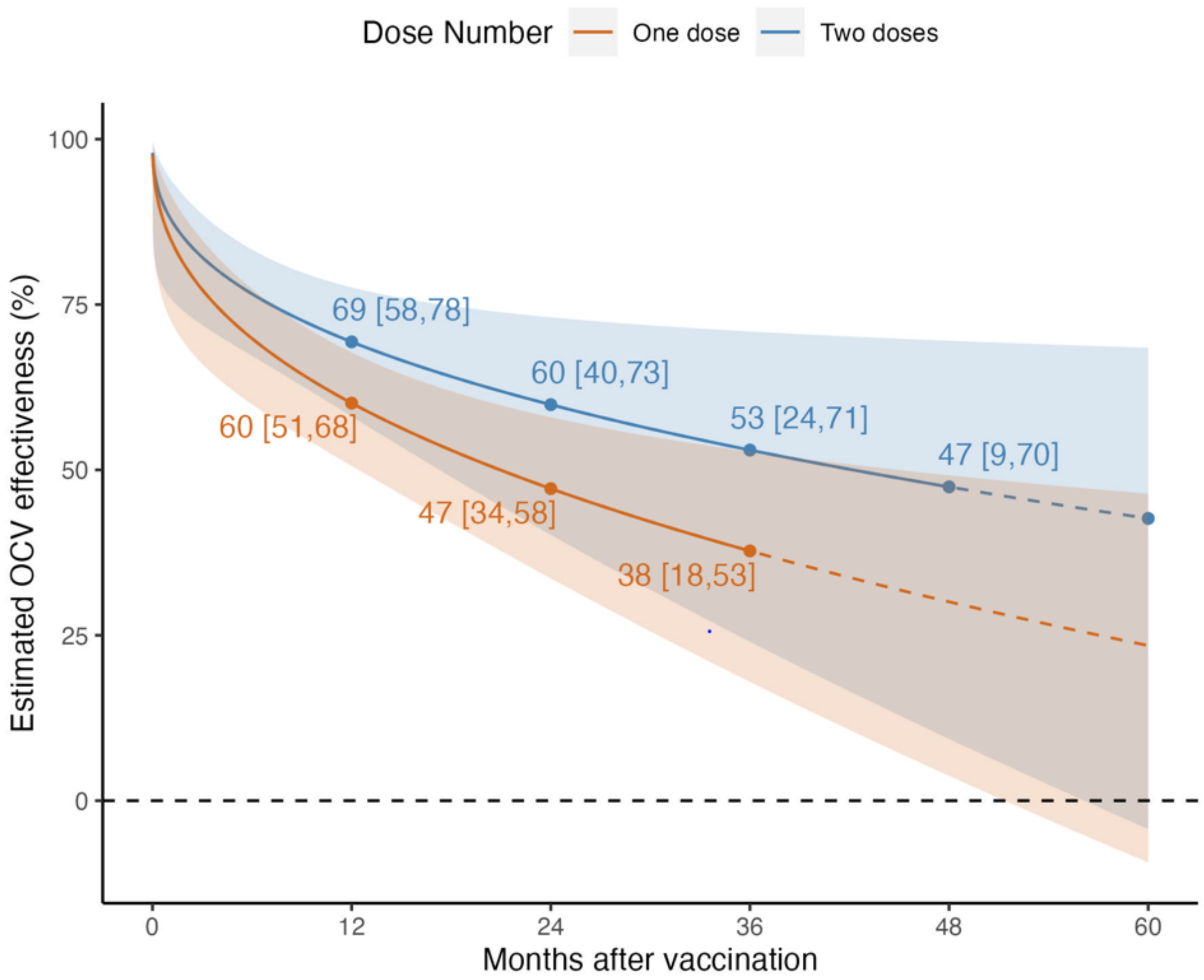

Figure S4. ***Comparison of estimated effectiveness of one-dose and two-dose kOCV over time since vaccination predicted by meta-regression models.*** *The orange and pale blue curves represent effectiveness over time of one-dose and two-dose kOCV predicted by the meta-regression models, with the shaded bands representing the 95% confidence intervals and 95% prediction intervals. The filled circles indicate the predicted estimates at 12, 24, 36, 48 (for two-dose estimate only) months post-vaccination, the value and 95% confidence interval is labelled below. The dashed horizontal line denotes no protective effect (0%) of kOCV. The dashed curves represent the extrapolated effectiveness for the follow-up period without any reported data from literature.*

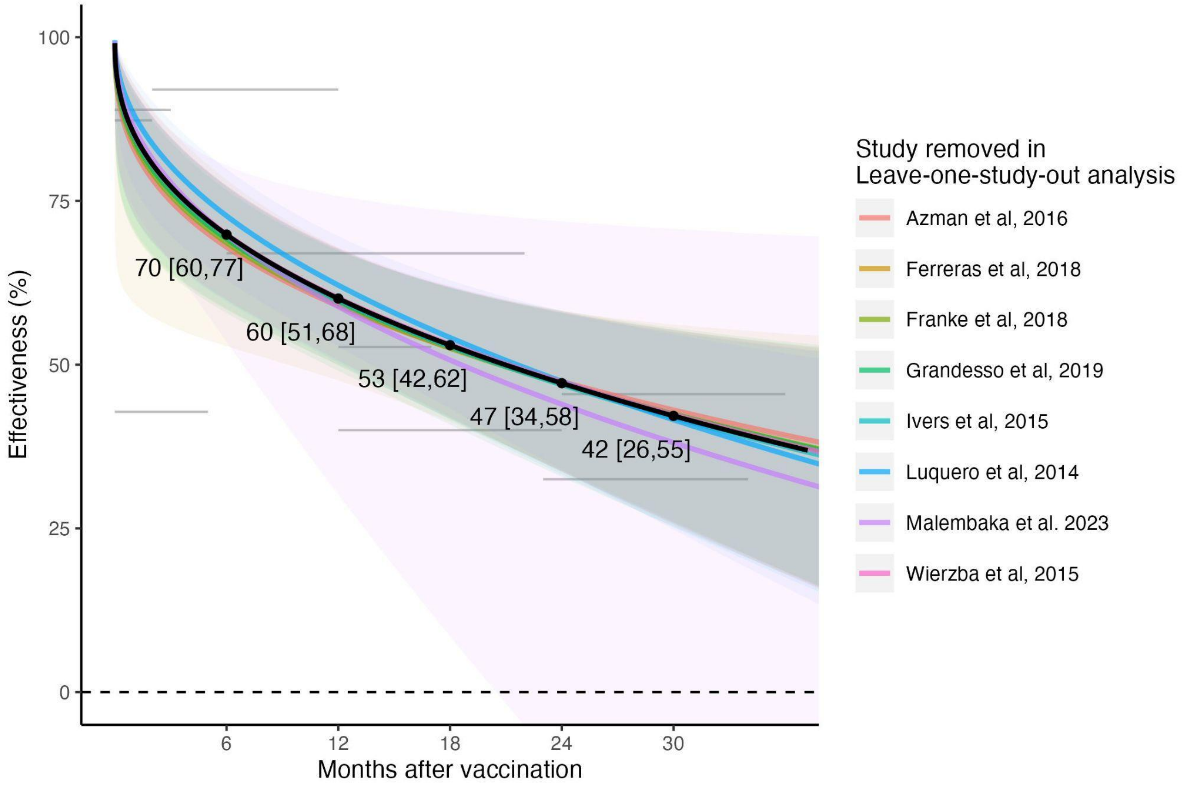

Figure S5. ***Effectiveness of one-dose kOCVs from leave-one-study-out analysis.*** *The black solid lines and labels are estimated efficacy or effectiveness using the full dataset of all two-dose estimates from included studies. The colored lines represent estimated efficacy and effectiveness after leaving out estimates from one study. The horizontal gray lines represent the full dataset that was used to fit the meta-regression models, the length of the line indicates the duration of follow-up (months since vaccination). The line's position on the y-axis marks the magnitude of the point estimate (%). The dashed horizontal line at y=0 denotes no protective effect (0%) of kOCV.*

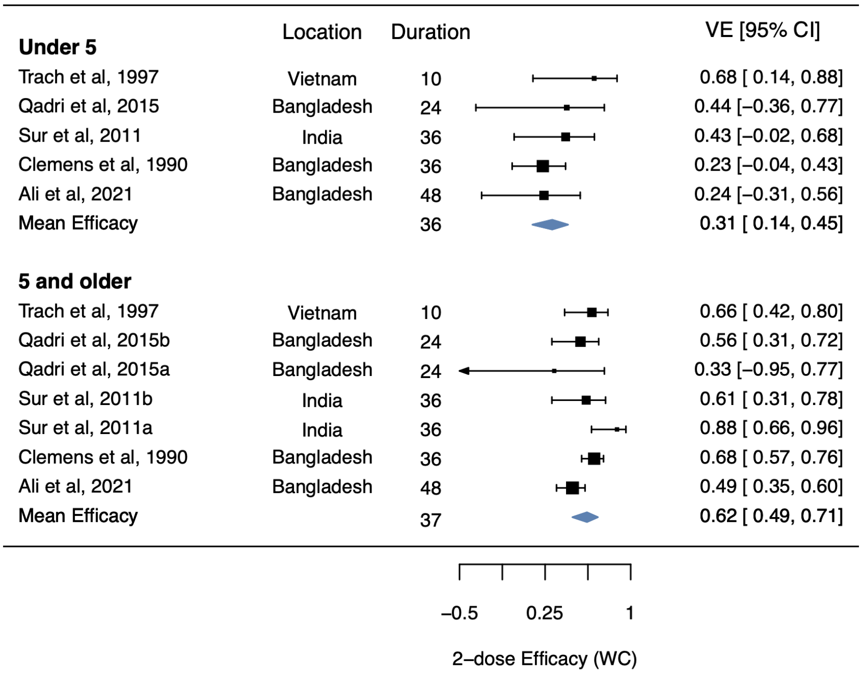

Figure S6. ***Pooled efficacy of two-dose whole-cell kOCV by age group.*** *Duration of the mean efficacy of each age group was weighted mean duration of the included estimates. Clemens et al. 1990 [19] was using three doses instead of two doses but is still included in the analyses. Sur et al. 2011a and Qadri et al. 2015a were subgroup estimates for participants aged between 5 and 15, while Sur et al. 2011b and Qadri et al. 2015b were subgroup estimates for participants aged above 15 years old [6,20]. The estimates included in this pooled analysis were efficacy estimates for the whole follow-up period. Black bars and squares show 95% confidence intervals and point estimates of efficacy for the studies. Blue diamonds show the pooled efficacy estimates for participants under 5 or 5 and older.*

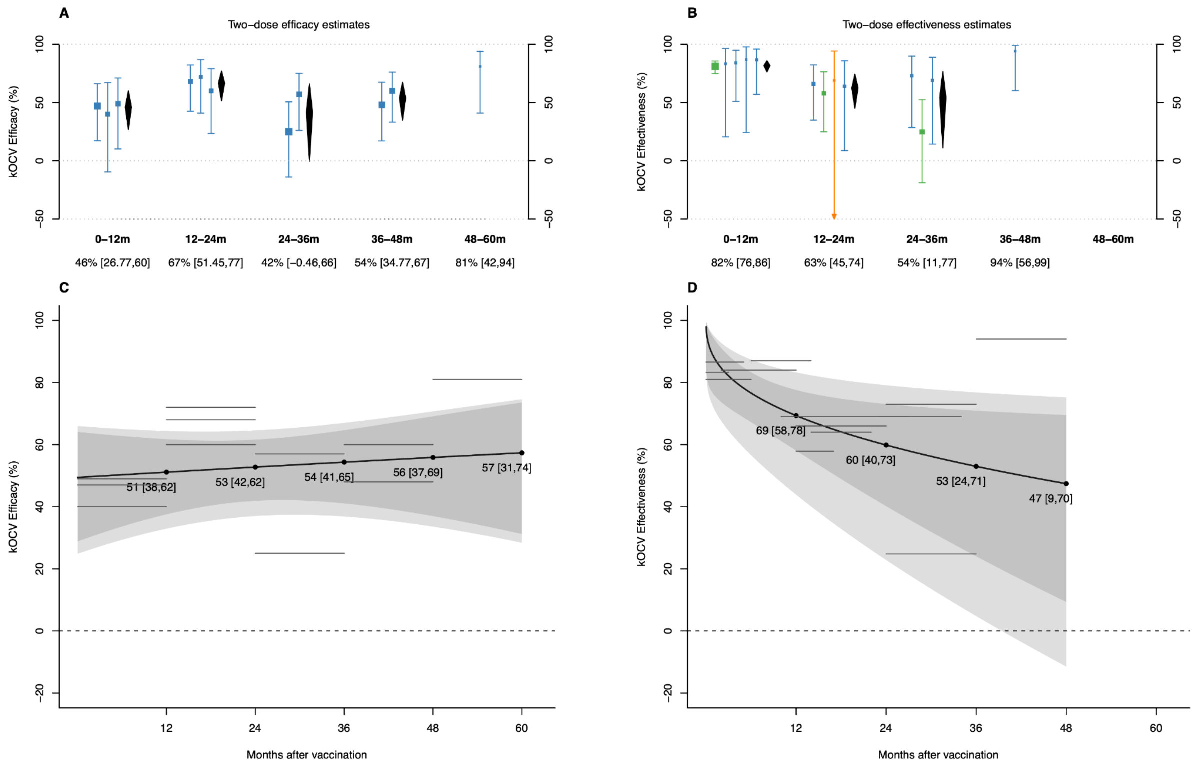

Figure S7. ***Stratified and meta-regression estimates of the efficacy and effectiveness of two doses of killed whole-cell OCV (kOCV) as a function of time since vaccination, after removing vaccines that came before Shanchol vaccines.*** *The upper panels illustrate stratified estimates of efficacy (A) and effectiveness (B) by time since vaccination bin (0-12, 12-24, 24-36, 36-48, 48-60 months after vaccination). The trial conducted in Matlab, Bangladesh [5] and the trial conducted in Hue, Vietnam [4] were not included in this figure and meta-regression analysis as they used pre-Shanchol vaccines. Estimates are grouped into the five follow-up duration categories by the midpoint of the time window during which the estimate was measured. Bars and squares show 95% confidence intervals (CI) and point estimates of efficacy or effectiveness for each literature, colored by vaccine type (blue: "Shanchol; green: Euvichol-plus; orange: Euvichol). Diamonds in black show the estimated average efficacy or effectiveness and 95% CI by follow-up period, with numerical values shown at the bottom of the x-axis in black. If there is only one estimate in the follow-up period, the estimate from the study is presented on the x-axis. The bottom panels illustrate meta-regression results for average two-dose (A) efficacy and (B) effectiveness as a function of time since vaccination, with the shaded envelope representing the 95% confidence intervals and 95% prediction intervals. The horizontal gray lines represent the data from the literature that were used to fit the meta-regression models, the length of the line indicates the duration of follow-up (months since vaccination). The line's position on the y-axis marks the magnitude of the point estimate (%). The dashed horizontal line at y=0 denotes no protective effect (0%) of kOCV.*

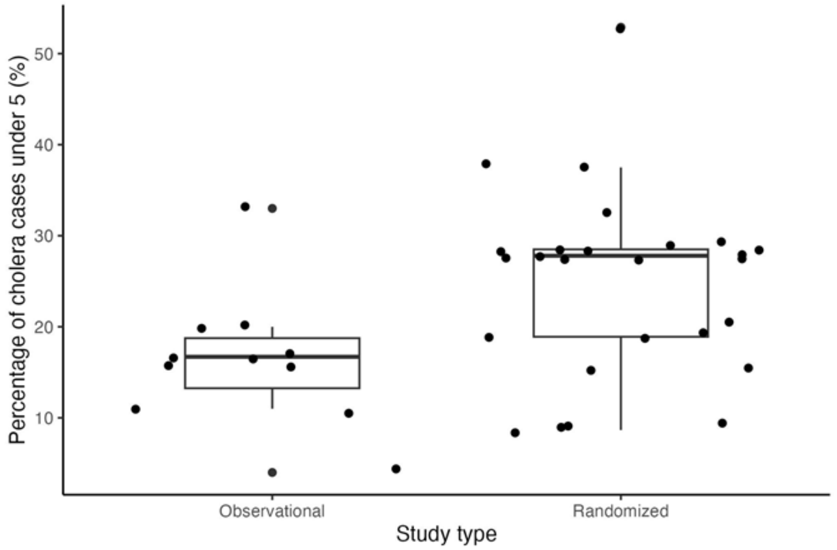

Figure S8. ***Distribution of percentage of cholera cases under 5 years old in efficacy studies and effectiveness studies.*** *Each point represents the percentage of cholera cases that are under 5 years old in one estimate. The box demarcates the 25th, 50th (median) and 75th percentile and the whiskers extend beyond the 15th and 75th percentiles by 1.5 times the interquartile range. The median percentage of cases under 5 is 16.7% for observational (effectiveness) studies and 27.8% for randomized (efficacy) studies.*

SUPPLEMENTARY REFERENCES

1. GA Wells, B Shea, D O'Connell, J Peterson, V Welch, M Losos, P Tugwell. The Newcastle-Ottawa Scale (NOS) for assessing the quality of nonrandomised studies in meta-analyses. Available: https://www.ohri.ca/programs/clinical_epidemiology/oxford.asp

2. Ali M, Qadri F, Kim DR, Islam MT, Im J, Ahmmed F, et al. Effectiveness of a killed whole-cell oral cholera vaccine in Bangladesh: further follow-up of a cluster-randomised trial. Lancet Infect Dis. 2021;21: 1407–1414.

3. Bhattacharya SK, Sur D, Ali M, Kanungo S, You YA, Manna B, et al. 5 year efficacy of a bivalent killed whole-cell oral cholera vaccine in Kolkata, India: a cluster-randomised, double-blind, placebo-controlled trial. Lancet Infect Dis. 2013;13: 1050–1056.

4. Trach DD, Clemens JD, Ke NT, Thuy HT, Son ND, Canh DG, et al. Field trial of a locally produced, killed, oral cholera vaccine in Vietnam. Lancet. 1997;349: 231–235.

5. van Loon FP, Clemens JD, Chakraborty J, Rao MR, Kay BA, Sack DA, et al. Field trial of inactivated oral cholera vaccines in Bangladesh: results from 5 years of follow-up. Vaccine. 1996;14: 162–166.

6. Qadri F, Ali M, Chowdhury F, Khan AI, Saha A, Khan IA, et al. Feasibility and effectiveness of oral cholera vaccine in an urban endemic setting in Bangladesh: a cluster randomised open-label trial. Lancet. 2015;386: 1362–1371.

7. Sialubanje C, Kapina M, Chewe O, Matapo BB, Ngomah AM, Gianetti B, et al. Effectiveness of two doses of Euvichol-plus oral cholera vaccine in response to the 2017/2018 outbreak: a matched case–control study in Lusaka, Zambia. BMJ Open. 2022;12: e066945.

8. Grandesso F, Kasambara W, Page A-L, Debes AK, M'bang'ombe M, Palomares A, et al. Effectiveness of oral cholera vaccine in preventing cholera among fishermen in Lake Chilwa, Malawi: A case-control study. Vaccine. 2019;37: 3668–3676.

9. Franke MF, Ternier R, Jerome JG, Matias WR, Harris JB, Ivers LC. Long-term effectiveness of one and two doses of a killed, bivalent, whole-cell oral cholera vaccine in Haiti: an extended case-control study. Lancet Glob Health. 2018;6: e1028–e1035.

10. Ivers LC, Hilaire IJ, Teng JE, Almazor CP. Effectiveness of reactive oral cholera vaccination in rural Haiti: a case-control study and bias-indicator analysis. The

Lancet Global. 2015. Available: https://www.sciencedirect.com/science/article/pii/S2214109X14703687

11. Luquero FJ, Grout L, Ciglenecki I, Sakoba K, Traore B, Heile M, et al. Use of Vibrio cholerae vaccine in an outbreak in Guinea. N Engl J Med. 2014;370: 2111–2120.

12. Matias WR, Guillaume Y, Augustin GC, Vissieres K, Ternier R, Slater DM, et al. Effectiveness of the Euvichol® oral cholera vaccine at 2 years: a case-control and bias-indicator study in Haiti. Int J Infect Dis. 2023. doi:10.1016/j.ijid.2023.11.022

13. Malembaka EB, Bugeme PM, Hutchins C, Xu H, Hulse JD, Demby MN, et al. Effectiveness of one dose of killed oral cholera vaccine in an endemic community in the Democratic Republic of the Congo: a matched case-control study. Lancet Infect Dis. 2024. doi:10.1016/S1473-3099(23)00742-9

14. Wierzba TF, Kar SK, Mogasale VV, Kerketta AS, You YA, Baral P, et al. Effectiveness of an oral cholera vaccine campaign to prevent clinically-significant cholera in Odisha State, India. Vaccine. 2015;33: 2463–2469.

15. Qadri F, Wierzba TF, Ali M, Chowdhury F. Efficacy of a single-dose, inactivated oral cholera vaccine in Bangladesh. England Journal of …. 2016. Available: https://www.nejm.org/doi/full/10.1056/nejmoa1510330

16. Qadri F, Ali M, Lynch J, Chowdhury F, Khan AI, Wierzba TF, et al. Efficacy of a single-dose regimen of inactivated whole-cell oral cholera vaccine: results from 2 years of follow-up of a randomised trial. Lancet Infect Dis. 2018;18: 666–674.

17. Ferreras E, Chizema-Kawesha E, Blake A, Chewe O, Mwaba J, Zulu G, et al. Single-Dose Cholera Vaccine in Response to an Outbreak in Zambia. N Engl J Med. 2018;378: 577–579.

18. Azman AS, Parker LA, Rumunu J, Tadesse F, Grandesso F, Deng LL, et al. Effectiveness of one dose of oral cholera vaccine in response to an outbreak: a case-cohort study. The Lancet Global Health. 2016;4: e856–e863.

19. Clemens JD, Sack DA, Harris JR, Van Loon F, Chakraborty J, Ahmed F, et al. Field trial of oral cholera vaccines in Bangladesh: results from three-year follow-up. Lancet. 1990;335: 270–273.

20. Sur D, Kanungo S, Sah B, Manna B, Ali M, Paisley AM, et al. Efficacy of a low-cost, inactivated whole-cell oral cholera vaccine: results from 3 years of follow-up of a randomized, controlled trial. PLoS Negl Trop Dis. 2011;5: e1289.
